# *In Vivo* Evaluation of a Subcutaneously Injectable Implant with a Low-Power Photoplethysmography ASIC for Animal Monitoring

**DOI:** 10.3390/s20247335

**Published:** 2020-12-21

**Authors:** Jose Manuel Valero-Sarmiento, Parvez Ahmmed, Alper Bozkurt

**Affiliations:** Department of Electrical and Computer Engineering, NC State University, Raleigh, NC 27695-7911, USA; jvalero@ncsu.edu (J.M.V.-S.); pahmmed@ncsu.edu (P.A.)

**Keywords:** injectable, photoplethysmography, biomedical electronics, biophotonics, encapsulation, implant, *in vivo*, biomedical telemetry, software radio

## Abstract

Photoplethysmography is an extensively-used, portable, and noninvasive technique for measuring vital parameters such as heart rate, respiration rate, and blood pressure. The deployment of this technology in veterinary medicine has been hindered by the challenges in effective transmission of light presented by the thick layer of skin and fur of the animal. We propose an injectable capsule system to circumvent these limitations by accessing the subcutaneous tissue to enable reliable signal acquisition even with lower light brightness. In addition to the reduction of power usage, the injection of the capsule offers a less invasive alternative to surgical implantation. Our current prototype combines two application-specific integrated circuits (ASICs) with a microcontroller and interfaces with a commercial light emitting diode (LED) and photodetector pair. These ASICs implement a signal-conditioning analog front end circuit and a frequency-shift keying (FSK) transmitter respectively. The small footprint of the ASICs is the key in the integration of the complete system inside a 40-mm long glass tube with an inner diameter of 4 mm, which enables its injection using a custom syringe similar to the ones used with microchip implants for animal identification. The recorded data is transferred wirelessly to a computer for post-processing by means of the integrated FSK transmitter and a software-defined radio. Our optimized LED duty cycle of 0.4% at a sampling rate of 200 Hz minimizes the contribution of the LED driver (only 0.8 mW including the front-end circuitry) to the total power consumption of the system. This will allow longer recording periods between the charging cycles of the batteries, which is critical given the very limited space inside the capsule. In this work, we demonstrate the wireless operation of the injectable system with a human subject holding the sensor between the fingers and the *in vivo* functionality of the subcutaneous sensing on a pilot study performed on anesthetized rat subjects.

## 1. Introduction

Photoplethysmography (PPG) is an optical measurement of blood volume that is obtained by shining light at a specific wavelength into the body and detecting the amount of light transmitted through, or backscattered from, the tissue [1]. PPG and pulse oximetry (an application of dual wavelength PPG) are extensively used to measure hemodynamic parameters such as heart rate, respiration rate, blood oxygen saturation, and even blood pressure [2,3]. PPG technology can be found in a variety of recent commercial products and form factors including finger clip sensors, smartwatches, smartphones, and fitness trackers, as it provides valuable health indicators in a continuous, convenient, and noninvasive way [4].

The deployment of PPG sensors in veterinary medicine presents a challenge due to the animal’s fur blocking most of the incident light where only a small fraction penetrates the tissue and reaches the photodetector [5]. Traditional clinical PPG and pulse oximetry measurements on animals require shaving hair or placing the sensors on the furless regions such as ear or tongue while constraining animal mobility due to required wiring. For that reason, these methods are only used in clinical settings by connecting clips to the ear or tongue of anesthetized animals [6]. One possible solution to overcome this barrier while maintaining a wearable form factor is to attach light pipes or optical fibers bundles to the light source to guide the light through the layer of fur and to increase the coupling with the skin [7], although this approach still requires higher current for the light source and is still relatively sensitive to motion artifacts. Another method to obtain good-quality optical signals is to enter the subcutaneous region and circumvent the layer of skin and fur in order to get a direct reading from the inner tissue. This has been studied and proposed in different ways, including reflective pulse oximetry systems surgically implanted in the neck area [8] or wrapped around an arterial blood vessel [2,9]. These prior works showed the potential of performing PPG measurements inside the body of an animal, although these still require an elaborate surgical operation.

We propose the use of an “injectable” system as a less invasive alternative to gain access to the subcutaneous tissue and improve the signal-to-noise ratio (SNR) of reflective PPG measurements. This would avoid the need for any surgical implantation procedure and free the animal from being tethered to a device. A similar injectable approach has been previously used for other applications in the literature, such as functional electric stimulation to reanimate paralyzed limbs in humans [10,11] or monitoring electrocardiogram-based heart rate, core body temperature, and gross motor activity in laboratory rats [12].

Towards that end, we had previously shown a preliminary feasibility analysis of the use of inductive coupling in powering a light-emitting diode (LED) that had been packaged in a glass tube with an inner diameter of less than 4 mm, and the capability of employing this small form-factor device to obtain PPG readings when held between the fingers of a human subject [13]. We now present the *in vivo* evaluation of an injectable capsule intended for the wireless monitoring of PPG in small animals. The capsule system includes a custom low-power analog front end (AFE) integrated into an application-specific integrated circuit (ASIC) for amplifying and reading the photocurrent generated from an off-chip photodiode (PD). This amplified photocurrent modulated by the hemodynamics of the blood vessels is used to extract the heart rate information. A microcontroller connected to the AFE generates the related control signals, serializes the output stream, and creates the data frame. Finally, an on-chip transmitter creates a binary frequency-modulated (FM) signal to wirelessly send the digital data stream outside the body. We employ a software-defined radio (SDR) to receive the data in a laptop and perform post-processing. To evaluate and demonstrate a proof of concept for our proposed system, we tested the integrity of the wireless subsystem in data transmission and the feasibility of an injectable probe in PPG data acquisition on two different setups with a human and a rat subject. Section 2 describes the details of the system design followed by its performance evaluation in Section 3. Section 4 discusses the methods and outcomes of the *in vivo* experiments. Finally, a discussion on future research directions in Section 5 is followed by the conclusion in Section 6.

## 2. System Architecture

**Figure 1:**
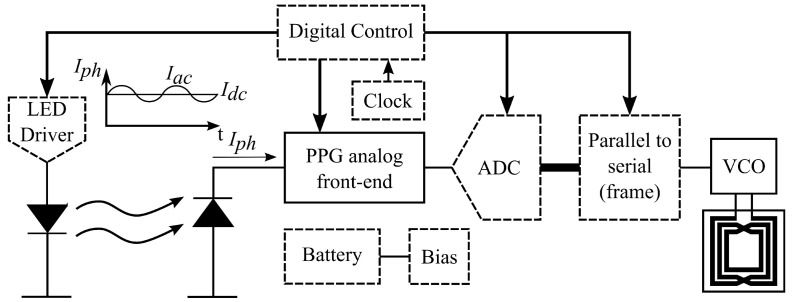
Block diagram of the subcutaneous PPG system where the blocks in solid boxes have been integrated using the ON Semiconductor 0.5-μm CMOS process and the blocks in dashed boxes have been implemented with smaller COTS components.

**Figure 2:**
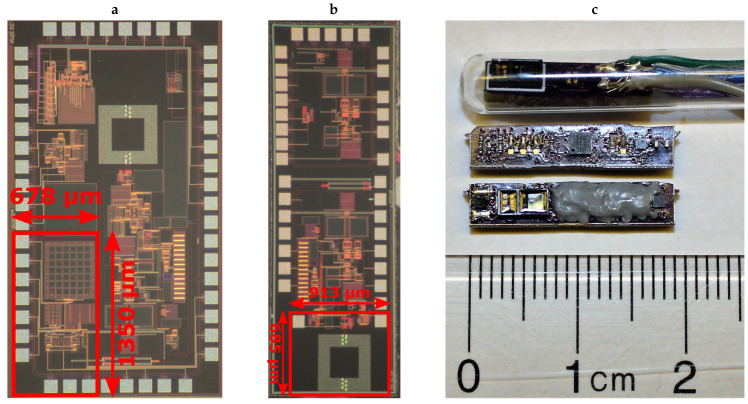
Micrographs of the two ASICs (before being wire bonded to the PCB) and a photo of the overall system. (**a**) The delimited die area measures approximately 1.3 × 0.7 mm^2^ and represents the transimpedance amplifier, current digital-to-analog converter, and switched integrator. (**b**) The delimited die area measures approximately 0.7 × 0.9 mm^2^ and represents the FSK transmitter. (**c**) Picture of the injectable optical probe with an OSRAM BioMon sensor in the glass encapsulation (top) and the two sides of the fully assembled injectable PPG system.

The various blocks that constitute the proposed injectable system are presented in Figure 1, where the solid blocks are parts of the ASICs fabricated using a 0.5-μm CMOS process (ON Semiconductor, Denver, CO, USA), and the dashed blocks are implemented with commercial off-the-shelf (COTS) components. For the current proof-of-concept prototype, we created a custom and miniaturized printed circuit board (PCB) to fit into an injectable glass capsule where the two different ASICs containing the PPG analog front end and the wireless transmitter are placed adjacent to each other on the same PCB. Figure 2a shows the photo of the die that contains the AFE within an active area of 0.9 mm^2^, while Figure 2b shows the photo of the second die with the transmitter occupying an active area of 0.6 mm^2^. The remaining building blocks use external COTS components with small footprints without compromising the target capsule size (Figure 2c). For injection, the largest size COTS needle we had access to is 2 gauge which allows for a capsule with an outer diameter of 6 mm. Considering the glass wall thickness, we targeted a PCB width of 3.2 mm. In terms of the length of the capsule, we were able to fit all the necessary components into a 19-mm long PCB layout which is a reasonable size for a injectable device intended for animal monitoring.

In this current version, we implemented the control logic using an ultra-small COTS microcontroller (Atmel^®^ ATtiny10, 2.0 × 2.0 × 0.6 mm^3^, Microchip Technology, Inc., Chandler, AZ, USA) which can actually fit into the limited volume of our implantable capsule. This is a low-power CMOS 8-bit microcontroller based on the AVR^®^ enhanced RISC architecture with 1024 Bytes of programmable flash program memory and internal 8-bit ADC [14]. The clock frequency can be scaled based on the supply voltage. In our case, we ran the system at 4 MHz with a supply of 2.5 V. The output of the AFE is directly connected to one of the IO lines of the microcontroller, which digitizes the signal using its internal 8-bit ADC, creates the data frame, and outputs a serial stream ready to be transmitted, thereby making the recorded PPG waveform available for visualization and post-processing in the receiver.

On the top side of the PCB, we soldered the ATtiny10 microcontroller along with the required passive components and an ultra-small bilateral analog switch (SN74LVC1G66, 1.0 × 1.0 × 0.4 mm^3^, Texas Instruments, Dallas, TX, USA) that briefly switches off the LED between sampling pulses and reduces the power consumption significantly. The bottom side of the PCB contains a 2.5 V capacitor-free low-dropout regulator (LDO) available in a small X2SON package (TLV733P, 1.0 × 1.0 × 0.4 mm^3^, Texas Instruments, Dallas, TX, USA). We included another IC containing a pair of N-channel MOSFETs in a leadless ultra-small and ultra-thin SMD plastic package (PMDXB600UNE, 1.1 × 1.0 × 0.37 mm^3^, Nexperia, Nijmegen, The Netherlands) to serve as current references for the AFE circuit. We chose an integrated solution for the LED and PD pair (SFH7050 BioMon Sensor, 4.7 × 2.5 × 0.9 mm^3^, OSRAM Opto Semiconductors GmbH, Regensburg, Germany), which includes one PD and three LEDs with different wavelengths (green at 525 nm, red at 660 nm, and infrared at 950 nm) along with a light barrier in between to minimize optical crosstalk with the PD. The BioMon sensor is also placed on the bottom side to ensure tissue contact. In order to fit the two ASICs within the dimensional constraints of the capsule PCB, we wire-bonded these directly to the board and covered with glob top epoxy (EPO-TEK^®^ T7109-19, Epoxy Technology, Inc., Billerica, MA, USA).

As mentioned earlier, the two system blocks that we designed and integrated into the two ASICs are: (1) the AFE for reading and conditioning the photocurrent from the PD; and (2) the frequency-shift keying (FSK) transmitter. The following subsections describe the design considerations and implementation techniques of the PPG signal conditioning, the control logic, and the FSK transmission in detail.

### 2.1. PPG Signal Conditioning

**Figure 3:**
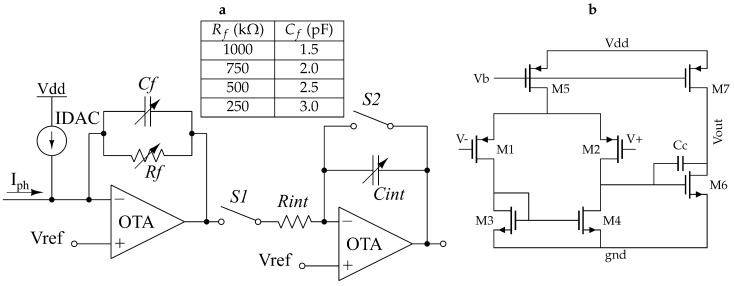
(**a**) Schematic of the PPG analog front end. The inset shows the programmable options for choosing among the built-in $R_{f}$-$C_{f}$ value pairs. (**b**) Transistor-level schematic of the OTAs used in the AFE.

The AFE conditions the PPG signal before being sampled and digitized. This process includes passing only the AC component of the photocurrent containing the heart rate information along with its amplification and conversion to voltage. The AFE is also used for reverse biasing the PD to make the acquisition process faster, by reducing the parasitic capacitance of the diode and thereby increasing its response speed. This method also introduces a new source of DC offset and noise error, which requires further considerations [15]. The readout circuit for the PPG signal is comprised of a transimpedance amplifier (TIA), a current digital-to-analog converter (IDAC), and a switched integrator (SI), as shown in Figure 3a. The TIA converts the current captured by the PD into a voltage. The IDAC cancels out a programmable portion of the the DC component of the photocurrent at the input of TIA. The output from the TIA is then further amplified and filtered with the SI before being sampled by the ADC.

In the reflection-mode PPG acquisition, the LED and PD are placed on the same side of the tissue as opposed to the transmission-mode [1]. Although, in our case, the PD is physically very close to the LED because of the size constraints, these are optically isolated by a built-in light barrier. The incident light on the PD generates an AC and DC photocurrent. The AC signal is caused by the changes in blood volume and is the signal of interest as it contains the dynamic physiological information such as heart rate or respiration rate. The slowly-varying DC component is contributed by the non-pulsatile part of the blood and other tissues, the presence of the ambient light, the leakage of light that is directly coupled from the LED despite the light barrier, and the dark noise. As the AC component is much smaller than the DC component (the ratio is usually less than 10% [16]), it is required to subtract the latter in order to avoid saturating the readout circuit and to increase the dynamic range. This is achieved by using the IDAC at the input of the TIA. In order to help the system adapt to different perfusion conditions when combined with a variable LED driver, the IDAC is designed with two control bits to select a current with a value of 1, 1.25, 1.5, or 1.75 times of a reference current ($I_{ref}$), which is an internally generated source of constant DC bias current. For experimental flexibility, we also included the capability to generate $I_{ref}$ externally in this prototype. We obtained the best results during the *in vivo* experiments by using 10 μA as $I_{ref}$. More control bits can be added to the IDAC to further expand the dynamic range.

The TIA converts the remaining input current into voltage along with a gain factor, while keeping the voltage across the diode constant and operating the PD in current mode. This provides better linearity for the system [15]. We implemented the TIA using an operational transconductance amplifier (OTA) as detailed in Figure 3b. The two-stage Miller-compensated OTA consists of a differential-to-single-ended amplifier followed by a common-source amplifier. We anticipated PD current values in the order of tens of nA for small animal measurements, so we implemented amplification factors as high as 10^6^ using a resistive feedback. We also included two control bits to tune the feedback resistor ($R_{f}$) from 250 kΩ to 1 MΩ in four-steps to accommodate the intrinsic variability of the PPG measurements. We also added a compensation capacitor ($C_{f}$) in parallel with the feedback resistor to introduce a left half-plane zero in order to prevent the instability caused by the feedback network in the TIA [17,18]. The same two bits that control the value of the resistor also select an appropriate pre-designed value for the capacitor (the $R_{f}$-$C_{f}$ value pairs has been tabulated in the inset of Figure 3a) so that the stability of the TIA is maintained across the four different combinations and the transfer function does not vary significantly.

The second stage in the readout circuit is an SI that adds extra gain to the signal path, and, more importantly, creates a low-pass filter (LPF) before the signal is sampled. This is required because the TIA creates a peak in the noise transfer function, so an LPF can counteract this effect and keep an acceptable signal-to-noise ratio [19]. This peaking is caused by the pole that the feedback resistor of the TIA creates with the input capacitance, usually dominated by the parasitic capacitance of the PD. The integrator is implemented using an RC circuit formed by $R_{int}$ and $C_{int}$ (Figure 3a), where the switched capacitor is placed in a feedback loop with another OTA with the same design as Figure 3b.

(1) $$V_{out}=\frac{T_{int}}{R_{int}\;\cdot \;C_{int}}\left(R_{f}\;\cdot \;I_{ph}\right)$$

**Figure 4:**
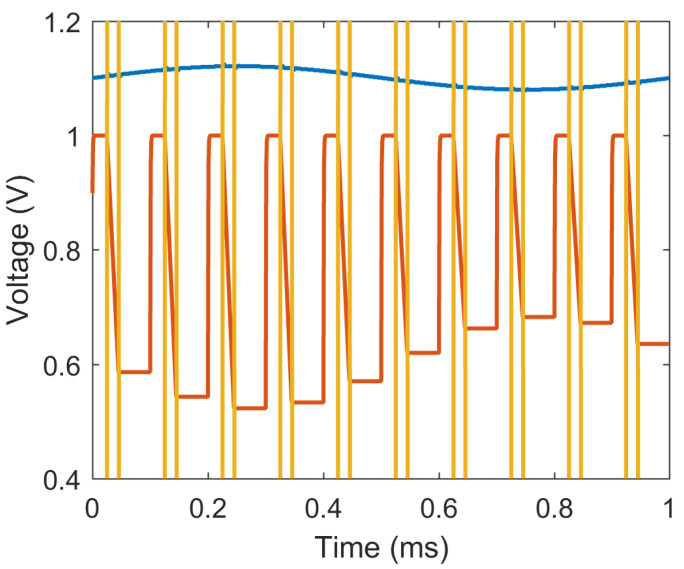
Simulation results of the PPG readout circuit. (Blue) Output of the transimpedance amplifier. (Orange) Output of the switched integrator. (Yellow) Control signal for switch S1, which is also used for the LED driver.

The total gain of the signal path can be described by Equation (1) where $R_{int}$ has a fixed value of 100 kΩ, and $C_{int}$ can be tuned between 25 pF and 100 pF in four-steps using two control bits. The integration time $T_{int}$ is determined by the on-time of switch S1, and is another parameter that we can use to set the gain of the readout circuit. A second control signal is required for switch S2 to discharge the feedback capacitor once the ADC samples the output signal. The waveform depicted in Figure 4 represents a snapshot of the simulated operation of the readout circuit, where the input photocurrent and the outputs of the TIA and SI are shown.

### 2.2. Control Logic

**Figure 5:**
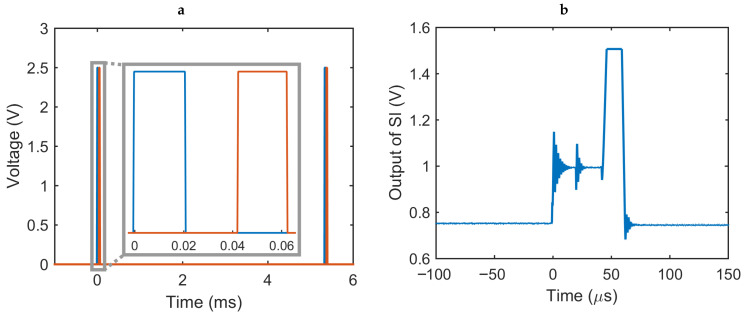
(**a**) The control signals generated by the microcontroller repeating every 5.3 ms. The inset shows SI_RST (blue) and SI_EN (orange) used for operating switches S2 and S1, respectively. (**b**) Measured output of the switched integrator showing the instant when switch S2 resets the feedback capacitor and switch S1 enables the integration of the photocurrent immediately after that.

The operation of the PPG readout circuit is orchestrated by three control signals: (1) SI_RST resets the SI by discharging the feedback capacitor and prepares the system for the next sample; (2) SI_EN is utilized to briefly turn the LED on and enable the SI through the switch S1 such that the amplified photocurrent can be captured; and (3) ADC_EN enables the ADC to start the conversion once the output of the integrator has settled. The most critical timing requirement among these signals is the pulse duration of SI_EN, represented as $T_{int}$, as it has a direct impact on the total gain of the circuit as well as the overall power consumption of the system. This duration is constrained by a lower bound governed by the settling time of the TIA, whereas the active time of the LED should be ideally reduced as much as possible to save energy. For our circuit, we chose an on-time of 20 μs (0.4% duty cycle) over a sampling frequency of approximately 200 Hz, as Figure 5a depicts.

The timer/counter module of the microcontroller is utilized as a timing reference for the control signals, generating an interrupt at every 20 μs and toggling the value of SI_RST or SI_EN appropriately (see the inset of Figure 5a). Figure 5b shows the measured output of the SI in response to these control signals. The third control signal, ADC_EN, is generated internally in the microcontroller. The main program loop takes the 8-bit output of the ADC and sends it to the VCO for wireless transmission through one of the IO pins after generating the data frame. The control logic generates the data frame by inserting a predefined header before the 8 bits of the sample, and creates the serial data stream with an effective burst rate of 5 kBd. The complete implementation of the control logic used 52% of the available program memory and less than 10% of the data memory in the microcontroller. The next iteration of the ASIC will replace this microcontroller with our implementation of the ADC and the control logic to lower the power consumption even further.

### 2.3. FSK Transmission

A voltage-controlled oscillator (VCO) is integrated as the last stage of our system to act as a binary FM transmitter and to generate an FSK signal. The resonant circuit consists of a 26 nH planar inductor and two accumulation-mode varactors that serve as the tuning elements. In order to minimize the bandwidth of transmission, the tuning voltage is reduced to 200 mV from the 2.5 V digital signal using a resistive divider. We presented the details of this circuit in [20] where it was used as an analog FM transmitter instead, i.e., the recorded signal modulated the varactors directly. In this application, we connected the serial data stream generated by the microcontroller to create the FSK signal. A predefined header byte precedes each byte of the data to help with the synchronization at the receiver.

**Figure 6:**
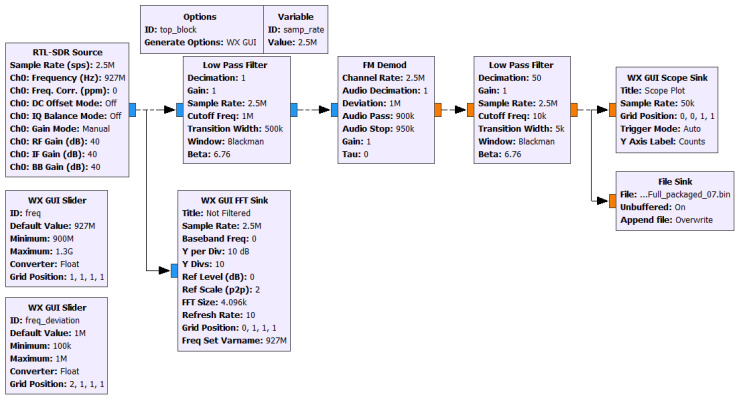
Screenshot of the GRC interface showing the different blocks that comprise the software-defined FSK receiver.

On the receiver side, we used a similar setup as the one described in [20], connecting a dipole antenna to an advanced SDR-based receiver (AirSpy R2 by AirSpy Networks, Inc., Paris, IDF, France) plugged into a Windows^®^ laptop running an open-source software development kit, GNU Radio. Figure 6 shows the flow graph of our custom FSK receiver developed using the GNU Radio Companion (GRC) tool. The first block is the data source representing the USB dongle with a complex output containing the baseband representation of the received FSK signal. This signal is passed through an LPF to limit its bandwidth to 1 MHz and fed into an FM demodulator with a frequency deviation that matches this limited bandwidth. The output is, then, passed through another LPF with a cutoff frequency of 10 kHz to extract our band of interest and reduce the noise. We used various time scopes and fast Fourier transform (FFT) sinks in the system to check the signal at different stages of the receiver, and a file sink to save the incoming stream of data so we can post-process it in MATLAB^®^. This binary file contains the raw time-domain signal at 50 ksps. In order to extract the actual 8-bit samples from the ADC, we first need to apply a binary slicer and obtain a sequence of binary symbols. Then, the actual samples can be extracted by recognizing the predefined header that we added to the data frame. Finally, we convert the digital samples to an analog scale to represent the voltage levels of the recorded PPG waveform.

## 3. Measurement of System Performance

**Figure 7:**
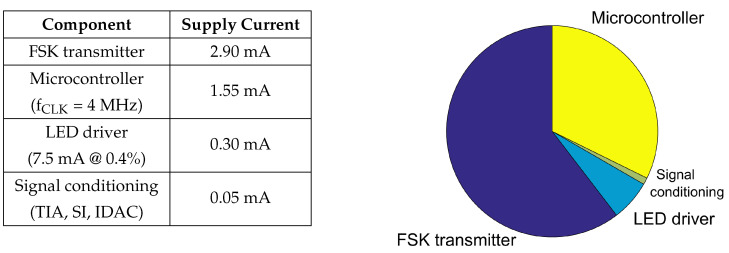
Distribution of power consumption drawn from a 2.5 V source by the PPG readout system.

The power consumption of a PPG system is dependent on the magnitude of the peak LED current that is required for a signal with sufficient SNR, i.e., the amount of light that can be effectively modulated by the changes in blood volume. However, this dependence is minimized by optimizing the duty cycle of our system to 0.4% at a sampling rate of 200 Hz. Consequently, the total power consumption of 12 mW (4.8 mA of supply current) from the regulated supply of 2.5 V is dominated by the FSK transmitter drawing 2.9 mA of current. This is followed by the microcontroller at 1.55 mA. The microcontroller operates at a frequency of 4 MHz using the calibrated internal 8 MHz oscillator, and keeps the timer/counter module and ADC continuously running. This power consumption could be further optimized by utilizing the sleep mode of the microcontroller between sampling events, although our objective is to completely integrate its functionality into a single ASIC in our next iteration and obtain better efficiency and compactness altogether. Based on our pulsing scheme, the LED contributes an average current of 0.3 mA in the default experimental setup, while the signal conditioning blocks would account for a current of 0.05 mA (see Figure 7 for a graphical representation).

We evaluated two different COTS pin-type non-rechargeable lithium batteries (BR425 and BR435 from Panasonic Corporation, Kadoma, Osaka, Japan) as the single-use power source. These are two of the options that can be easily purchased and used in injectable form factor. The nominal capacity of these batteries (25 and 50 mAh in BR425 and BR435, respectively) is expected to allow for 5–10 h of continuous operation. However, the actual performance was not on par due to the peak current values that were higher than the nominal discharge rates (0.5 or 1.0 mA, respectively) and the apparent inability of these particular batteries to sustain short-but-high discharge pulses at this rate [21]. However, a smaller and rechargeable pin battery has been recently announced by the same company (CG-320B) with discharge characteristics that exceed our current requirements [22]. It has not been made commercially available yet, as of the publication of this paper, but we believe that it will enable further miniaturization of our system in the future. While we had shown the feasibility of leveraging the wireless power transfer in [13], further research for wireless power transfer is underway and is beyond the scope of this paper.

We measured the input-referred noise of the AFE by placing the test board of our ASIC inside a Faraday cage, recording segments of 50 s (64,516 samples) of the SI output with an oscilloscope, and estimating its power spectral density (PSD) by calculating the FFT in MATLAB^®^. In order to obtain the input current noise at different values of photocurrent and measure the effect of the shot noise of the PD, we placed the optical probe containing the LED and PD pair inside a opaque dark box, to minimize any external light interference and control the amount of incident light by tuning the LED driver. We also measured the actual gain of the circuit by comparing the change in the output when applying a step of 100 nA at the input with a setup of $R_{f}$ = 250 kΩ, $C_{f}$ = 3.0 pF for the TIA and $C_{int}$ = 25 pF for the SI, resulting in a value of approximately 2 MΩ as expected from Equation (1).

**Figure 8:**
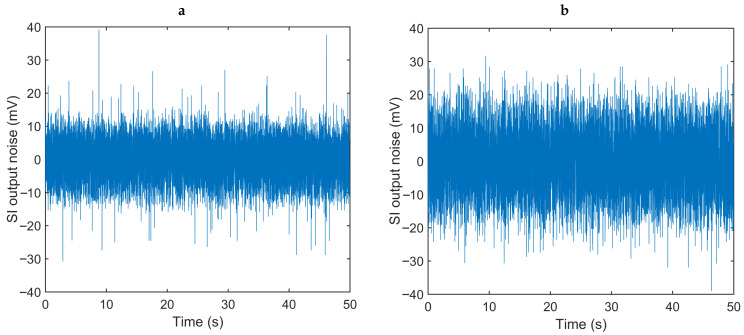
Noise at the output of the switched integrator for (**a**) a photocurrent of 3 μA, and (**b**) a photocurrent of 10 μA, showing the effect of the PD shot noise.

The timing of the control signals has a direct impact on the performance of the circuit, because the integration time affects the AFE gain. Therefore, as our current implementation generates these signals through a microcontroller, the inherent jitter of the SI_EN signal has a major contribution on the noise of the readout circuit. Another main contributor to the noise of the AFE is the shot noise of the PD. Its effect is shown in Figure 8 by comparing the noise at a photocurrent of 3 μA and 10 μA, representing an increase of 590 pA_RMS_ in the integrated current noise. Table 1 shows a comparison of our readout circuit with some other integrated PPG front ends, presented previously in the literature or available commercially. Our readout circuit provides a reasonable noise performance with low power consumption and comparable active area, which facilitates the integration of the complete system inside an injectable capsule. The noise could be further minimized once the digital core is embedded in the same ASIC and the jitter of the control signals is reduced. The readout circuit we present achieves a satisfactory duty cycle that directly benefits the power consumption of the system, as the high peak current of the LED is ON for a very short fraction of the sampling period. This will enable longer recording periods between battery charging cycles, which is critical given the very limited space available within the glass tube.

**Figure 9:**
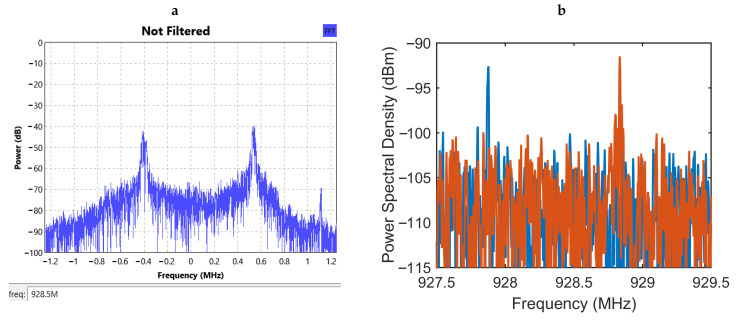
(**a**) Relative power spectral density at the front end of the SDR receiver. (**b**) Two superposed plots of power spectral density measurements recorded with a real-time spectrum analyzer to obtain the absolute levels of the received signal.

In order to evaluate the performance of the wireless communication system, the dipole antenna was placed 60 cm away from the capsule and connected to the AirSpy R2 SDR receiver. Figure 9a shows the relative PSD recorded at the front end of the receiver. As the GNU Radio operates with many different hardware interfaces that are not strictly calibrated, obtaining the absolute power of the received signal [27] was not possible. However, we utilized a real-time spectrum analyzer (Model 3066, Tektronix, Inc., Beaverton, OR, USA) to record the absolute power levels. Figure 9b shows the superimposed plots of two such PSD measurements.

**Figure 10:**
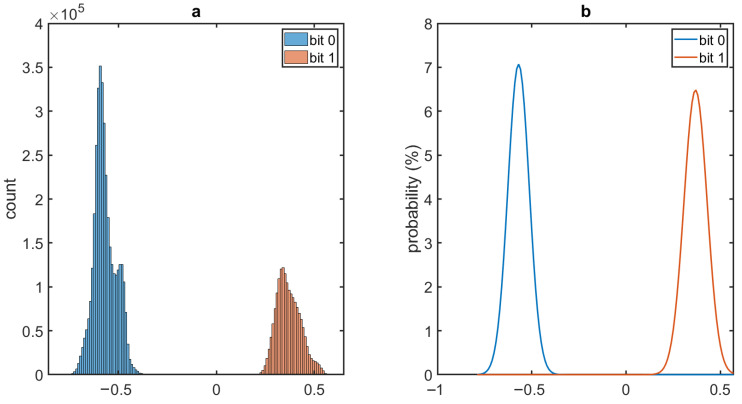
(**a**) Histograms of received binary signal levels showing the actual distribution for the symbols ‘0’ and ‘1’. (**b**) Gaussian fit for probability density functions ($P_{0}$ and $P_{1}$) of the received signal levels for the two symbols.

To further investigate the quality of the communication link, we estimated the bit error rate (BER) of the received signal. BER of a binary signal is defined as the conditional probability of receiving an erroneous bit and expressed as,

(2) $$\mathrm{BER}=\frac{1}{2}P_{0}\left(X>V_{th}\right)+\frac{1}{2}P_{1}\left(X<V_{th}\right)$$

where $P_{0}$ and $P_{1}$ are the probability density functions (PDFs) of the received signal levels for the symbols ‘0’ and ‘1’ respectively and $V_{th}$ is the digitization threshold. For this purpose, we have analyzed $5.4\times 10^{6}$ sample points to fit Gaussian curves to the PDFs of the symbols separated by $V_{th}=0$ (Figure 10). The calculated BER is $3.16\times 10^{-10}$ which is fairly low. This is expected because of the larger gap between the signal levels of the two symbols.

## 4. *In Vivo* Experimental Results

**Figure 11:**
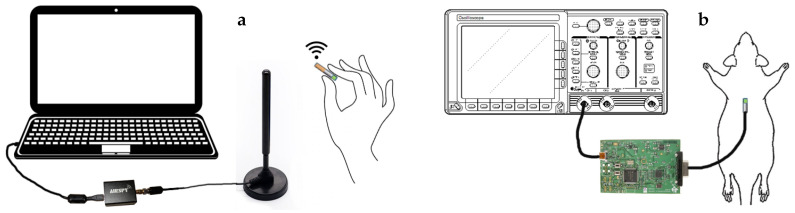
Visual description of the two experimental setups. (**a**) Wireless transmission was tested with the handheld capsule transmitting PPG data to the laptop through the dipole antenna and the Airspy R2 receiver. (**b**) *In vivo* performance was tested on an anesthetized rat with the optical probe injected in the thoracic area. The probe was connected to the ASIC test board and the AFE4490 evaluation board alternatively and the data was displayed on an oscilloscope.

**Figure 12:**
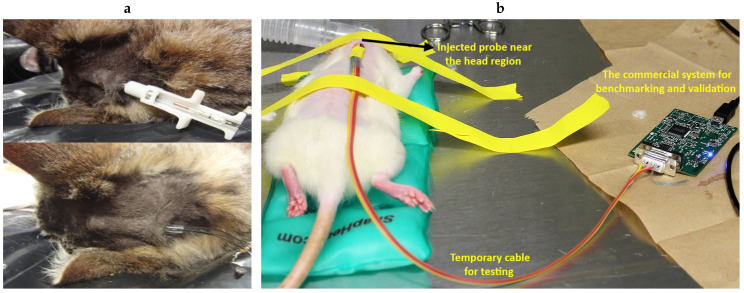
(**a**) Subcutaneous injection procedure demonstrated with a cat cadaver. (The picture has been republished from [13] with authors’ permission ©2014 IEEE.) (**b**) An anesthetized rat with the subcutaneously injected optical probe temporarily wire-connected to the commercial AFE4490 system for benchmarking and validation.

**Figure 13:**
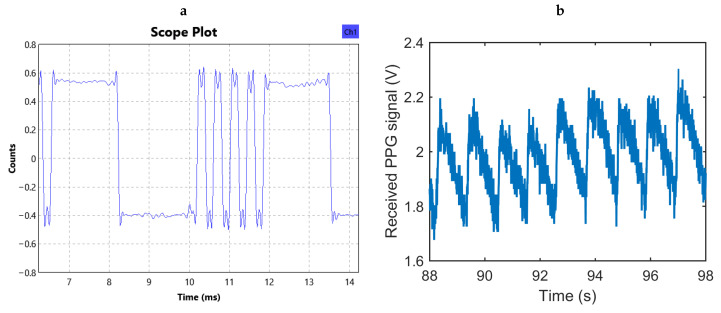
(**a**) An excerpt of the received data stream after being demodulated and low-pass filtered in the SDR. (**b**) Received PPG waveform collected from the human finger after extracting the 8-bit samples from the serial data stream.

For proof of concept, we performed two *in vivo* experiments as shown in Figure 11 to test the functionality of the integrated system and the feasibility of using the injectable probe in obtaining PPG signals with adequate quality (Figure 12). In the first part of the study, we tested the functionality of the completely assembled system along with the battery and used the finger of a human subject to generate a representative PPG pulse for a quick assessment. Although the system was designed for subcutaneous applications and on animals, this setup provided a simpler access to a real PPG signal for initial testing. The system depicted in Figure 2c was placed inside a glass tube that the subject held during the measurement with the index finger touching the surface of the glass and facing the photodetector and thumb pressing on the opposite site for adequate contact. We used the maximum gain of the readout circuit and an LED peak current of 7.5 mA. An excerpt of the received signal after being demodulated and low-pass filtered in the SDR is shown in Figure 13a, and the equivalent analog representation of the received samples after the post-processing is shown in Figure 13b. This plot clearly demonstrates the PPG waveform and identifies the heart rate being approximately 66 bpm as also verified by a gold standard COTS PPG system (CMS50D+ Pulse Oximeter, Contec Medical Systems Co., Ltd., Qinhuangdao, Hebei, China).

**Figure 14:**
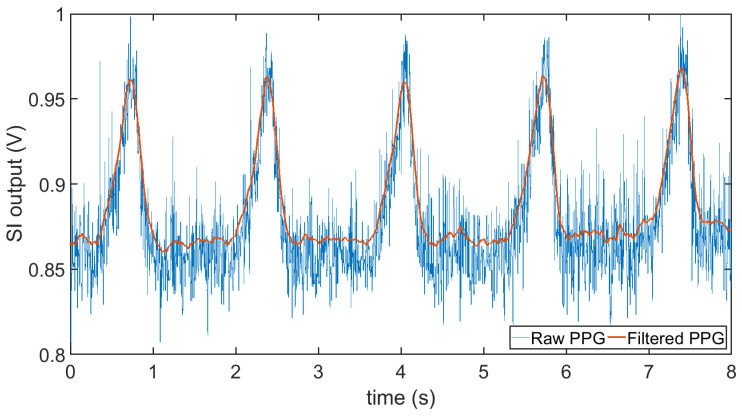
The raw signal from the output of the switched integrator and the filtered signal after performing a moving average when using the presented PPG readout circuit during the rat experiment demonstrating a more pronounced respiratory pattern and buried heart beats.

In the second experiment, we used an anesthetized rat (*Rattus norvegicus*) to evaluate the performance of the subcutaneous PPG sensor. All animal procedures took place in the College of Veterinary Medicine at NC State University and were approved by the Institutional Animal Care and Use Committee (IACUC) of NC State University. The rat was first anesthetized using a controlled flow of isoflurane. For experimental flexibility, we subcutaneously injected the glass capsule containing only the OSRAM BioMon sensor (the top capsule in Figure 2c) at several locations of the rat’s body using a COTS metal needle attached to a 3D-printed injector with Acrylonitrile Butadiene Styrene (ABS) plastic (Figure 12a). For benchmarking, we connected it alternatively with our test board and a commercially available evaluation module for pulse oximetry (AFE4490SPO2EVM, Texas Instruments, Dallas, TX, USA). The test board included the ASIC with the signal conditioning circuit, the LED driver, and the ATtiny10 microcontroller. In this experiment, we directly connected the output of the SI to an oscilloscope (DSOX3014A, Keysight Technologies, Santa Rosa, CA, USA) to get rapid feedback on the signal quality. The whole experiment lasted for about an hour (10–15 min of data collection from each of the body locations). Figure 14 shows the result that we were able to obtain from the thoracic area on the back of the rat (Figure 12b) using the maximum gain setup in our readout circuit, with $R_{f}$ = 1 MΩ, $C_{f}$ = 1.5 pF in the TIA, and $C_{int}$ = 25 pF in the integrator, accounting for a total gain of 8 MΩ. The respiration pattern can be clearly seen whereas the external noise has visually masked the light modulation from the blood volume changes caused by the heart contraction and made the heart rate estimation challenging.

**Figure 15:**
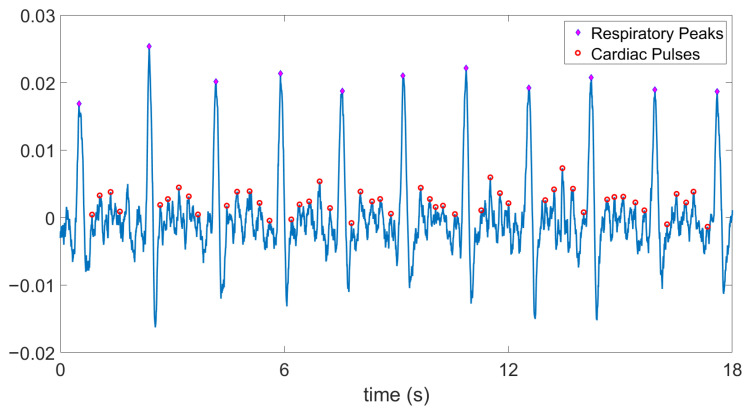
Digital post-processing of the PPG signal from the ASIC output demonstrates discernible heart beats. The mean ± SD of the averaged heart rate values in between each pair of respiratory events is 241 ± 18 bpm and that of respiration rate is 35 ± 1.45 breaths per minute.

In order to clean up the noisy PPG signal, we have used a moving-average filter with a 3-dB cutoff frequency at 6 Hz. As it is fundamentally difficult to record a simultaneous “ground-truth” signal for such optical measurements, we calculated the SNR of the PPG signal using this filtered signal (the red waveform in Figure 14) as the ground-truth to get more insight on the noise. We found the SNR to be 5.14 dB which is very low. Furthermore, the large respiratory artifacts made it difficult to detect the periodicity of the heart pulses even from the filtered signal. Hence, we applied another moving-average filter with a 3-Hz cutoff frequency on the raw PPG signal to obtain the baseline for the respiratory signal. Subtraction of these two signals partially removed the respiratory artifacts. Finally, after these post-processing steps, we performed peak detection on the cleaner PPG signal (Figure 15). The short-term average of the heart rate values were calculated using the peaks in between each pair of respiration events. The mean ± SD of these averaged values was found to be 241 ± 18 bpm.

**Figure 16:**
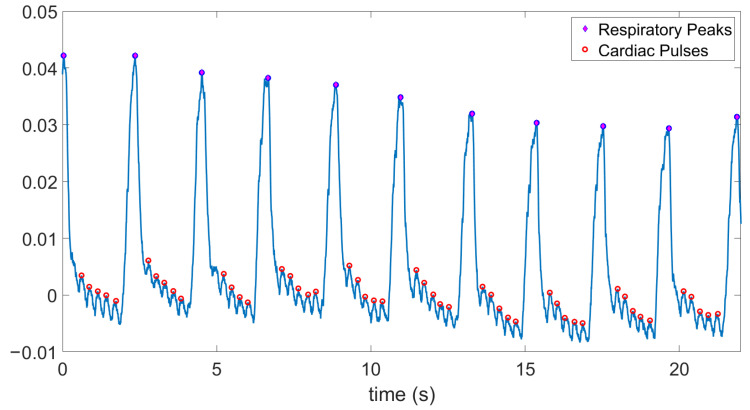
Signal from the AFE4490 evaluation board connected to the probe injected in the anesthetized rat. The respiratory pattern and heart contraction related modulations can be clearly seen. The mean ± SD of the averaged heart rate values in between each pair of respiratory events is 225 ± 6 bpm and that of respiration rate is 27 ± 1.20 breaths per minute.

**Figure 17:**
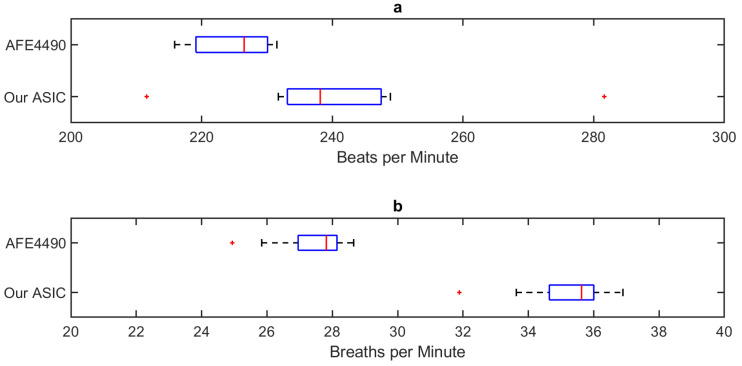
Whisker plot of the extracted (**a**) heart rate and (**b**) respiration rate values consecutively obtained using the presented ASIC and COTS AFE4490. The measured values were in the expected range as verified by alternative methods performed by veterinarians. The blue boxes, the red lines within the boxes, and the red markers outside denote the interquartile ranges, their medians, and the outliers (based on the 1.5 IQR rule) respectively.

For validation and comparison purposes, we connected the same optical probe to the AFE4490 evaluation board and obtained the representative results shown in Figure 16 with a gain setup of 10 kΩ. We observe the same respiration pattern that we had previously obtained, but in this case, the heart-beat generated variations were more visible. The mean ± SD of the averaged heart rate values in between each pair of respiration events was calculated to be 225 ± 6 bpm. Although the standard deviation of the heart rate values extracted from our system is higher than that of the AFE4490, the interquartile ranges are similar in both cases excluding a few outliers (Figure 17).

## 5. Future Work

Several areas of improvement remain to further address the circuit integration challenges in such a miniaturized injectable implant. Firstly, the two circuit blocks implemented in the two ASICs can be combined monolithically in a future version of the ASIC. Secondly, as an intermediate step to test the performance of the system and for experimental flexibility, the presented system used a COTS microcontroller to implement the control logic and data conversion. We have already implemented an on-chip ADC in one of the ASICs using a successive-approximation register (SAR) topology for lower power consumption. It samples the output voltage of the conditioning block and creates a parallel stream of 8 bits of data. It was difficult to utilize this ADC in the current system, because miniaturized COTS microcontrollers do not offer sufficient number of input/output (IO) ports required for accessing the 8-bit ADC output. However, we plan to leverage this on-chip ADC design in the next iteration of the ASIC where we will also integrate the logic for the control signals in order to completely remove the external COTS microcontroller.

Moreover, we observed a very low SNR with the existing design in the *in vivo* experiments. One of the main differences between our custom ASIC and the commercial solution is the differential-to-single-ended versus the fully-differential implementation, which removes most of the systematic noise. The next iteration of our ASIC is expected to reduce this noise and eliminate the need for post-processing. Furthermore, we have only evaluated non-rechargeable batteries in this work due to the unavailability of rechargeable batteries in this form factor. A reasonable next-step is to integrate the wireless charging capability for sustainable use of the implant in animal monitoring. Finally, the current topology is designed for PPG readings where only one wavelength of the LEDs is utilized. However, it could be adapted to perform pulse oximetry by including another LED driver for the second wavelength, changing the control logic accordingly to multiplex the same AFE, and allocating the data from both LEDs in the output stream.

## 6. Conclusions

The use of traditional clinical photoplethysmography or pulse oximetry in veterinary medicine has been limited to the ear and tongue regions of anesthetized animals. The deployment of wearable PPG technology in freely-moving animals has been hindered by the challenges presented by the thick layer of skin and fur on the effective transmission of light through tissue. In this paper, we presented our efforts towards the development of a fully wireless injectable capsule for continuous monitoring of heart rate and respiratory rate in animals through the use of subcutaneous PPG. Our injectable solution opens up the possibility of obtaining PPG readings from animals outside of the clinical settings with the potential benefit of lower power requirement thanks to lower LED peak currents needed as opposed to having the LED and PD pair applied externally and directly against the hair. An injectable solution also offers a less invasive alternative to surgical insertion which is a similar injection mechanism as currently used for animal identification using microchip implants.

For experimental flexibility, our current implementation comprises two different ASICs, one containing the PPG readout circuit and the other FSK transmitter. Our plan is to combine these monolithically in a single IC in the next iteration. The signal conditioning circuit is comprised of a transimpedance amplifier and a switched integrator, to amplify and filter the photocurrent that is generated in the PD while operating with a pulsing LED to reduce power consumption. It also has a current DAC to cancel out most of the DC component from the PPG signal and increase the dynamic range of the sensor. A small microcontroller generates the control signals for the readout circuit, digitizes the output of the switched integrator, and creates the serial stream of data for the FSK transmitter. The current system fits inside a 40 mm long glass tube with an inner diameter of 4 mm, enabling subcutaneous injection using a custom 3D-printed syringe. Finally, we implemented a software-defined radio to receive, demodulate, and post-process the PPG waveform in a laptop.

We provided a preliminary demonstration of the functionality of our system by performing *in vivo* experiments in anesthetized rats, obtaining respiration and heart rate readings by using our readout circuit. We validated the feasibility of this injectable system in subcutaneous PPG monitoring of small animals by means of an injectable sensor probe and comparing the collected waveforms with the results obtained with an evaluation board of a large-size COTS analog front end IC. We also demonstrated the wireless transmission of data through the integrated FSK transmitter and the SDR receiver when recording PPG from a human subject holding the fully-assembled capsule between fingers for demonstration purpose. We are in the process of integrating most of the external components into one single chip to further minimize the size and power consumption.

## Figures and Tables

**Table 1:** Comparison of readout circuit performance to other integrated PPG front ends.

Literature	This Work	TBCAS ’08 [23]	TBCAS ’10 [24]	TBCAS ’15 [25]	TBCAS ’13 [19]	AFE4490 [26]
Technology	0.5 μm	0.35 μm	1.5 μm	0.18 μm	0.35 μm	NR *
Supply Voltage	2.5 V	2.5 V	5.0 V	1.8 V	3.3 V	2–3.6 V
Sample Rate	200 Hz	100 Hz	100 Hz	165 Hz	100 Hz	20–5000 Hz
DC Current Rejection	17.5 μA	43.9 μA	NR	100 μA	NR	10 μA
Integrated Noise	1 nA_RMS_	3.53 nA_RMS_	NR	600 pA_RMS_	NR	50 pA_RMS_
Noise Bandwidth	10 Hz	6 Hz	5 Hz	10 Hz	10 Hz	20 Hz
Noise Photocurrent	3 μA	NR	NR	30 μA	NR	5 μA
Active Area	0.92 mm^2^ ${}^{1}$	0.55 mm^2^	NR	0.84 mm^2^	1.15 mm^2^	NR
Power Consumption ${}^{2}$	125 μW${}^{1}$	600 μW	400 μW	128 μW	363 μW	2.1 mW
LED Duty Cycle	0.4%	10%	3%	0.7%	3–7%	1–25%

^1^ Includes TIA, SI, and IDAC. ^2^ Analog readout circuit only, excluding the reported power used by any digital core or LED driver. * NR—Not reported.
